# Harnessing hybrid edge-fog computing framework for emergency medical response management

**DOI:** 10.1097/MS9.0000000000004495

**Published:** 2025-12-04

**Authors:** Ehtisham Haider, Muhammad Umer Farooq Mujahid, Sakarie Mustafe Hidig

**Affiliations:** a Punjab Medical College, Faisalabad, Pakistan; b University of Health Sciences, Lahore, Pakistan; c Research Centre, Hargeisa Group Hospital, Hargeisa, Somalia

*Dear Editor*,

Healthcare systems in modern times rely on centralized cloud platforms, which show poor scalability in terms of latency. Delays in milliseconds can be fatal in case of a medical emergency, which is unacceptable when patient vitals are to be monitored or resources vital to patients are needed immediately. To eliminate the latency and bandwidth bottlenecks and connectivity breakdowns caused by remote cloud platforms, we need to switch to decentralized Hybrid Edge-Fog Computing systems^[1]^. This model is not just a modest upgrade to the current emergency healthcare management system but a paradigm shift towards a resilient, next-generation healthcare management system. This letter is prepared and presented in accordance with the TITAN 2025 guidelines^[2]^.

Although there is an increasing body of literature in cloud and IoT-based healthcare systems, the majority of existing methods depend on static offloading and a centralized control, which reduces flexibility and resilience in cases of high load or failure-prone conditions^[1,3]^. The role of computationally distributed across a heterogeneous node dynamically with ultra-low latency and reliability during medical emergencies with little literature has been studied by only a few studies. This is a major gap in research and implementation in the contemporary healthcare systems^[3]^.

The Hybrid Edge-Fog framework spreads the computation to three levels: the edge (wearables, bedside monitors, and IoMT devices) to filter and collect data; the fog nodes (hospital gateways, ambulance servers, and local medical stations) to perform real-time analytics and make decentralized decisions; and the central cloud to store data, manage electronic health records, and train AI models. It has three important use cases showing its clinical impact. First, there is a great enhancement of real-time monitoring of patients. The edge devices pass only essential data, including irregular heart rates and oxygen levels, or emergency alerts, saving up to sixty-percent of bandwidth as compared to raw data^[4]^. Second, it is possible to optimize resource allocation and ambulances, ventilators, and medical staff management, on a local level. Fog nodes algorithms’ load balancing is done in real-time when large cohort loads of patients are involved, as it is also scalable and reliable. Third, the model improves situational awareness in hospital networks because it processes data that is near to points of care, thus providing quicker decision support and automated clinical alerts^[3,4]^.

Such performance can be achieved through adaptive optimization and fine security. Experiments on simulated systems show that compared to cloud-only ones, edge-fog systems based on hybridization can provide a reduction in latency (66.67 times), energy efficiency (9% percent higher), and reliability (96%) of node failures, which are important measurements in mission-driven healthcare^[4]^. The Mog cloud resource management taxonomy also emphasizes the conservation of energy, scheduling, reliability, and scalability as the key aspects regarding the maintenance of real-time medical performance. Hybrid edge-fog networks are also decentralized which present some further security issues. A system based on Blockchain (BC), Software-Defined Networking (SDN), and Zero-Trust (ZT) will increase data integrity, provide transparent traceability, and decrease the Average Task Response Time (ART) by approximately 40−1 and Data Integrity (DI) by 30−1 and proves that a secure system and tremendously low latency can exist in healthcare environments^[5]^.

Research and policy assistance are needed to make the transition to this architecture. The next step in the work is Adaptive and Learning-based Task Placement with Deep Reinforcement Learning (DRL) to predictive offloading to get additional latency improvements in critical care. Tele-surgery, mobile ICUs and connected ambulances will be key in integrating with 5G/6G and Ultra-Reliable Low-Latency Communication (URLLC). To test these systems in the actual hospital settings, policymakers, technology providers, and healthcare leaders should focus on the creation of standardized, interoperable testbeds (with the help of tools such as iFogSim or FogNetSim++) to test them. To fill this gap, there is need to apply experimental work which bridges the gap between theoretical models and the deployable interoperable systems that have been tested under real emergency conditions^[6]^.

To conclude, Hybrid Edge-Fog Computing is a viable route to safer, faster, and more reliable delivery of healthcare – an inevitable place for contemporary emergency medicine. Its introduction can change the circulation of valuable information between patients, clinicians, and response teams and ensure that the life-saving alternative is decided based on the latest and most pertinent information that can be created. The continuum of this technology as being associated with the engineers, clinicians, and policymakers is a prerequisite to operationalize the innovation regarding the long-term, sustainable healthcare systems.
